# Supplementary material for: Effects of IFIH1 rs1990760 variants on systemic inflammation and outcome in critically ill COVID-19 patients in an observational translational study
Source: eLife. 2022 Jan 21;11:e73012. doi: 10.7554/eLife.73012 (PMC8782569; doi:10.7554/eLife.73012)
Supplement: Supplementary file 1. — 1a. Primers and probes used to detect SARS-CoV-2. 1b: Primers used for qPCR of human genes. 1c: Clinical characteristics of patients according to IFIH1 rs1990760 genotypes and dexamethasone therapy. Values are shown as absolute count or median (interquartile range). PBW: Predicted body weight. NIV: Non-invasive ventilation. PEEP: Positive End-Expiratory Pressure. *p-values calculated for proportion over the number of intubated patients. [file elife-73012-supp1.docx]

***IFIH1* rs1990760 variants, systemic inflammation and outcome in critically-ill COVID-19 patients: an observational translational study**

Laura Amado-Rodríguez, Estefanía Salgado del Riego, Juan Gómez de Oña, Inés López-Alonso, Helena Gil-Peña, Cecilia López-Martínez, Paula Martín-Vicente, Antonio López-Vázquez, Adrián González-López, Elías Cuesta-Llavona, Raquel Rodríguez-García, José Antonio Boga, Marta Elena Álvarez-Arguelles, Juan Mayordomo-Colunga, José Ramón Vidal-Castiñeira, Irene Crespo, Margarita Fernández, Loreto Criado, Victoria Salvadores, Francisco José Jimeno-Demuth, Lluís Blanch, Belén Prieto, Alejandra Fernández-Fernández, Carlos López-Larrea, Eliecer Coto, Guillermo M Albaiceta

**Online data supplement**

**Supplementary File 1a.** Primers and probes used to detect SARS-CoV-2.

| **Design** | **Position** | **Name** | **Sequence (5´-3´)** | **Gen** |
| --- | --- | --- | --- | --- |
| In house | Sense primer | CoV-2-OVI-S | ATCAAGTTAATGGTTACCCTAACATGT |  |
|  | Antisense primer | CoV-2-OVI-A | AACCTAGCTGTAAAGGTAAATTGGTACC | ORF1ab |
|  | Probe MGB FAM | CoV-2-OVI-FAM | CCGCGAAGAAGCTA |  |
| CDC^1^ | Sense primer | 2019-nCoV_N1-F | GACCCCAAAATCAGCGAAAT |  |
|  | Antisense primer | 2019-nCoV_N1-R | TCTGGTTACTGCCAGTTGAATCTG | Gen N |
|  | Probe MGB VIC | 2019-nCoV_N1-P-VIC | CCGCATTACGTTTGGT^2^ |  |

^1^ Sequences obtained from https://www.cdc.gov/coronavirus/2019-ncov/lab/rt-pcr-panel-primer-probes.html.

^2^ Probe sequence has been shortened as it is a MGB probe

**Supplementary File 1b.** Primers used for qPCR of human genes.

| **Gene** | **Forward** | **Reverse** |
| --- | --- | --- |
| ***STAT1*** | 5'-CCGTTTTCATGACCTCCTGT-3' | 5'-TGAATATTCCCCGACTGAGC-3' |
| ***STAT3*** | 5'-TTTCACTTGGGTGGAGAAGG-3' | 5'-GCTACCTGGGTCAGCTTCAG-3' |
| ***FOXO3*** | 5'-ACAAACGGCTCACTCTGTCC-3' | 5'-TCTTGCCAGTTCCCTCATTC-3' |
| ***IL6*** | 5'-GGTACATCCTCGACGGCATCT-3' | 5'-GTGCCTCTTTGCTGCTTTCAC-3' |
| ***GAPDH*** | 5'-TCGGAGTCAACGGATTTGGTCGT-3' | 5'-TGCCATGGGTGGAATCATATTGGA-3' |

**Supplementary File 1c.** Clinical characteristics of patients according to *IFIH1* rs1990760 genotypes and dexamethasone therapy. Values are shown as absolute count or median (interquartile range). PBW: Predicted body weight. NIV: Non-invasive ventilation. PEEP: Positive End-Expiratory Pressure. *p values calculated for proportion over the number of intubated patients.

|  | No steroids | | Steroids | |  |
| --- | --- | --- | --- | --- | --- |
| Rs1990760 ► | CC/CT | TT | CC/CT | TT | P value |
| Demographics | | | | | |
| Age (y) | 68 (59 - 73) | 64.5 (59 - 68) | 66 (57 - 75) | 70 (63 - 76) | 0.209 |
| Sex  Male  Female | 30  5 | 8  6 | 97  30 | 39  12 | 0.204 |
| Race  Black  White  Latino  Asian | 0  34  1  0 | 0  13  1  0 | 2  112  12  1 | 1  48  1  0 | 0.746 |
| Body mass index (Kg/m^2^) | 28 (25 - 33) | 32 (29 - 34) | 30 (27 - 33) | 31 (26 - 34) | 0.566 |
| Days since symptom onset | 7 (7 – 10) | 10 (6 – 11) | 8 (6 – 11) | 8 (6 -10) | 0.857 |
| Days from hospital admission to ICU admission | 1 (0 - 3) | 1 (1 – 3) | 2 (1 – 4) | 2 (1 – 3) | 0.262 |
| APACHE-II score | 17 (13 – 20) | 17 (12.5 – 19) | 14 (11 – 17) | 15 (13 – 18) | 0.756 |
| Comorbidities | | | | | |
| Arterial hypertension | 20 | 10 | 74 | 28 | 0.77 |
| Diabetes | 9 | 4 | 28 | 10 | 0.87 |
| Chronic kidney disease | 4 | 0 | 7 | 5 | 0.577 |
| COPD | 3 | 1 | 7 | 5 | 0.735 |
| Cirrhosis | 0 | 0 | 1 | 1 | 0.761 |
| Neoplasms  No  Active  Past | 29  4  2 | 13  1  0 | 125  3  0 | 48  2  0 | 0.009 |
| Lung function at ICU admission | | | | | |
| Ventilation at admission  Spontaneous / NIV  Controlled invasive ventilation  Pressure support ventilation | 6  29  0 | 4  10  0 | 21  106  0 | 7  42  1 | 0.525 |
| FiO_2_ | 0.5  (0.4 - 0.6) | 0.45  (0.4 - 0.6) | 0.5  (0.4 - 0.6) | 0.5  (0.4 - 0.6) | 0.069 |
| PaO_2_/FiO_2_ (mmHg) | 189 (148 - 237) | 180 (102 - 231) | 220 (171 - 297) | 188 (144 - 223) | 0.006 |
| PaCO_2_ (mmHg) | 42 (39 - 47) | 43 (42 - 45) | 43 (39 - 48) | 44 (38 - 47) | 0.795 |
| Respiratory rate (min^-1^) | 18 (16 - 20) | 19 (18 - 21) | 18 (16 - 22) | 18 (16 - 20) | 0.834 |
| Arterial pH | 7.36  (7.32 - 7.4) | 7.39  (7.36 - 7.42) | 7.37  (7.32 - 7.42) | 7.38  (7.33 - 7.4) | 0.712 |
| Tidal volume/PBW (ml/Kg) | 7.3 (6.9 - 8.1) | 8.4 (7.9 - 9.2) | 7.5 (6.9 - 8.2) | 7.8 (6.7 - 9.2) | 0.134 |
| Plateau pressure (cmH_2_O) | 25 (22 - 29) | 27 (26 - 29) | 23 (21 - 27) | 24 (20 - 29) | 0.2 |
| PEEP (cmH_2_O) | 14 (10 - 16) | 14 (12 - 14) | 12 (10 - 14) | 12 (10 - 14) | 0.417 |
| Driving pressure (cmH_2_O) | 12 (10 - 14) | 14 (12 - 14) | 12 (10 - 14) | 12 (10 - 14) | 0.728 |
| Respiratory system compliance (ml/cmH_2_O) | 39 (32 - 53) | 36 (31 - 43) | 42 (32 - 50) | 37 (31 - 46) | 0.471 |
| Laboratory results | | | | | |
| Leukocytes (x10^3^/μl) | 8.20  (5.56 – 10.42) | 6.90  (5.94 – 9.68) | 8.33  (6.14 – 11.44) | 9.35  (6.01 – 11.68) | 0.836 |
| Lymphocytes (x10^3^/μl) | 0.68  (0.59 – 0.90) | 0.79  (0.58 – 12.67) | 0.66  (0.48 – 0.94) | 0.57  (0.45 – 0.91) | 0.028 |
| Serum creatinine (mg/dl) | 0.92  (0.68 - 1.41) | 0.81  (0.58 - 1.05) | 0.77  (0.61 - 1.06) | 0.87  (0.64 - 1.24) | 0.104 |
| Serum ferritin (ng/ml) | 1582  (1062 - 2576) | 645  (327 - 1025) | 1125  (757 - 1896) | 1015  (712 - 1389) | 0.004 |
| D-dimer (ng/ml) | 1098  (776 - 2351) | 1095  (717 - 1522) | 1026  (659 - 2026) | 1085  (633 - 1806) | 0.513 |
| Additional treatments at ICU admission | | | | | |
| Vasoactive drugs | 26 | 9 | 61 | 28 | 0.042 |
| Invasive mechanical ventilation | 34 | 12 | 117 | 47 | 0.561 |
| Neuromuscular blocking agents | 19 | 3 | 50 | 27 | 0.097* |
| Prone ventilation | 24 | 4 | 63 | 28 | 0.117* |
| ECMO | 1 | 0 | 2 | 2 | 0.735* |
| Outcomes |  |  |  |  |  |
| ICU length of stay (days)  Overall  Survivors | 19 (13 - 33)  15 (13 – 24) | 13 (10 - 16)  13 (10 – 16) | 13 (9 - 31)  12 (8 – 29) | 15 (10 - 22)  17 (8 – 22) | 0.451  0.620 |
| ICU mortality | 8 | 0 | 27 | 18 | 0.034 |
| Hospital mortality | 9 | 0 | 27 | 19 | 0.020 |
