# Supplementary material for: Effects of IFIH1 rs1990760 variants on systemic inflammation and outcome in critically ill COVID-19 patients in an observational translational study
Source: eLife. 2022 Jan 21;11:e73012. doi: 10.7554/eLife.73012 (PMC8782569; doi:10.7554/eLife.73012)
Supplement: Transparent reporting form [file elife-73012-transrepform1.docx]

***eLife’s* transparent reporting form**

We encourage authors to provide detailed information *within their submission* to facilitate the interpretation and replication of experiments. Authors can upload supporting documentation to indicate the use of appropriate reporting guidelines for health-related research (see [EQUATOR Network](http://www.equator-network.org/%20)), life science research (see the [BioSharing Information Resource](https://biosharing.org/)), or the [ARRIVE guidelines](http://www.plosbiology.org/article/info:doi/10.1371/journal.pbio.1000412) for reporting work involving animal research. Where applicable, authors should refer to any relevant reporting standards documents in this form.

If you have any questions, please consult our Journal Policies and/or contact us: [editorial@elifesciences.org](mailto:editorial@elifesciences.org).

**Sample-size estimation**

- You should state whether an appropriate sample size was computed when the study was being designed
- You should state the statistical method of sample size computation and any required assumptions
- If no explicit power analysis was used, you should describe how you decided what sample (replicate) size (number) to use

Please outline where this information can be found within the submission (e.g., sections or figure legends), or explain why this information doesn’t apply to your submission:

Given the exploratory nature of the study objective and the absence of previous data, no formal sample size calculations were performed. All patients admiited to one of the participant ICUs with confirmed SARS-CoV-2 infection and meeting the Kigali modification of ARDS criteria from 16-Mar-2020 to 10-Dec-2020 (corresponding to the first two waves of the pandemic) were included in the study. This has been described in the methods section of the manuscript.

**Replicates**

- You should report how often each experiment was performed
- You should include a definition of biological versus technical replication
- The data obtained should be provided and sufficient information should be provided to indicate the number of independent biological and/or technical replicates
- If you encountered any outliers, you should describe how these were handled
- Criteria for exclusion/inclusion of data should be clearly stated
- High-throughput sequence data should be uploaded before submission, with a private link for reviewers provided (these are available from both GEO and ArrayExpress)

Please outline where this information can be found within the submission (e.g., sections or figure legends), or explain why this information doesn’t apply to your submission:

Sample sizes are reported in the manuscript, figures and legends. No outlier was removed. Inclusion and exclusion criteria are given in the methods section. RNAseq data has been uploaded to GEO (accession numbers GSE168400 and GSE177025) and is freely available.

**Statistical reporting**

- Statistical analysis methods should be described and justified
- Raw data should be presented in figures whenever informative to do so (typically when N per group is less than 10)
- For each experiment, you should identify the statistical tests used, exact values of N, definitions of center, methods of multiple test correction, and dispersion and precision measures (e.g., mean, median, SD, SEM, confidence intervals; and, for the major substantive results, a measure of effect size (e.g., Pearson's r, Cohen's d)
- Report exact p-values wherever possible alongside the summary statistics and 95% confidence intervals. These should be reported for all key questions and not only when the p-value is less than 0.05.

Please outline where this information can be found within the submission (e.g., sections or figure legends), or explain why this information doesn’t apply to your submission:

Statistical methods can be found in the last paragraph of the methods section of the manuscript. Raw data, median, 1^st^ and 3^rd^ quartiles, and largest/smallest values no further than 1.5*IQR from the hinge are shown in figures. Statistical tests and sample sizes are reported in each figure legend. Exact p values are reported in figures and the manuscript.

(For large datasets, or papers with a very large number of statistical tests, you may upload a single table file with tests, Ns, etc., with reference to sections in the manuscript.)

**Group allocation**

- Indicate how samples were allocated into experimental groups (in the case of clinical studies, please specify allocation to treatment method); if randomization was used, please also state if restricted randomization was applied
- Indicate if masking was used during group allocation, data collection and/or data analysis

Please outline where this information can be found within the submission (e.g., sections or figure legends), or explain why this information doesn’t apply to your submission:

Due to the observational nature of the study, treatments were not randomized. This is clarified in methods section.

**Additional data files (“source data”)**

- We encourage you to upload relevant additional data files, such as numerical data that are represented as a graph in a figure, or as a summary table
- Where provided, these should be in the most useful format, and they can be uploaded as “Source data” files linked to a main figure or table
- Include model definition files including the full list of parameters used
- Include code used for data analysis (e.g., R, MatLab)
- Avoid stating that data files are “available upon request”

Please indicate the figures or tables for which source data files have been provided:

Due to privacy concerns, patients’ raw data cannot be released. Raw data for figures 3,4 and 5 are available as supplementary CSV files. Code for the in-silico clinical trials is available at <https://github.com/Crit-Lab/IFIH1_simulation>, and simulation details are given in the supplementary results file.
